# Delineating Factors Surrounding Emergency Dental Access Behavior for Nontraumatic Dental Conditions Among Patients With Available Access to Local Preventive Dental Care

**DOI:** 10.1155/ijod/9509544

**Published:** 2026-06-24

**Authors:** Ingrid Glurich, Aloksagar Panny, Neel Shimpi, Mary Dorsch, Arun Bhatta, Richard Berg, Amit Acharya, Gregory Nycz

**Affiliations:** ^1^ Marshfield Clinic Research Institute (MCRI), 1000 North Oak Avenue, Marshfield, Wisconsin, USA; ^2^ Methodist Health System: Methodist Dallas Medical Center, 1441 North Beckley Avenue, Dallas, Texas, USA; ^3^ Family Health Center of Marshfield Inc. (FHC-M), 1307 N Street, Joseph Avenue, Marshfield, Wisconsin, USA; ^4^ University of Minnesota School of Dentistry, 515 Delaware Street SE, Minneapolis, Minnesota, USA; ^5^ Aurora Research Institute, 3075 Highland Parkway, Downers Grove, Illinois, USA; ^6^ Aurora Health, 960 N 12th Street, Milwaukee, Wisconsin, USA

## Abstract

**Objectives:**

A case–control study examined factors contributing to emergency dental clinic access for nontraumatic dental conditions (ED‐NTDC) among individuals residing within 20 miles of 10 networked dental clinics comprising a dental safety net.

**Materials and Methods:**

Phase I: using dental diagnostic codes for ED‐NTDC visits and dental prophylaxis visits that occurred from January 1, 2019 through May 19, 2019, interrogation of an integrated medical‐dental electronic health record (EHR) identified cases (*n* = 1784) and controls (*n* = 18,612), respectively, matched by dental center. Unadjusted odds ratios (ORs) with 95% confidence intervals (CIs) and chi‐squared *p*‐values were determined using unadjusted bivariate logistic regression analysis for each demographic variable by comparing subgroup‐specific proportions of cases and controls to a designated reference subgroup. Phase II: survey tools were designed and targeted to Phase I cases (*n* = 84) and controls (*n* = 113). Applying descriptive statistics, variability identified between case and control survey question responses defined additional potential candidate variables contributing to risk for ED‐NTDC visits.

**Results:**

Statistically significant risk variables ORs were graphically depicted in a forest plot and included: age 18–35 (OR 1.4), 36–50 (OR 1.2), sex (male: OR 1.2), race (non‐White: (OR 1.7)), payer status (self‐pay: (OR 2.4); Medicaid: (OR 1.6), sliding fee: (OR 1.8)), and E‐NTDC visits in medical (OR: 2.8) or dental (OR 2.0) settings in the prior 2 years. Twice‐annual dental visit rates were higher among controls vs. cases. Cases vs. controls more frequently indicated: (1) a preference for pulling painful teeth; (2) dental visits were only necessary for dental emergencies; (3) delay in seeking dental care for emergent dental pain; (4) higher rates of dental fear. Economic factors, (“could not afford dental care” and “lack of insurance”) especially in younger age tiers, strongly contributed to ED‐NTDC visits.

**Conclusions:**

Barriers to oral health access for vulnerable populations persist. Public health initiatives targeting dental health maintenance and literacy require expansion.

## 1. Introduction

The lack of dental access remains a substantive contributing factor driving medical emergency visits for the subset of the population experiencing nontraumatic dental conditions (NTDC). Whereas the Affordable Care Act improved medical care access for many adults in the USA, provision for dental coverage is lacking. In the absence of oral prophylaxis, oral diseases may ensue and culminate in acute painful exacerbation. Lacking access to affordable dental care, patients facing dental emergencies seek immediate interventional care in medical emergency settings where symptomatic care targeting pain palliation and reduction of infectious processes, potentially including antibiotic treatment, is rendered. However, emergency care providers generally lack training and diagnostic resources to optimally address the genesis of the dental condition.

Public health and government agencies report that while curtailment of rising trends seen in inappropriate urgent care or emergency room (ER) visits for NTDC settings has gained some traction between 2010 and 2019, the average cost of such visits between 2014 and 2019 also increased by 62% [1]. The decline in escalating rates of NTDC access in ERs in Wisconsin was attributed to the establishment of dental safety net operations [2]. Center for Medicare and Medicaid Services (CMS) data were most frequently billed for NTDC ER visits, prompting expansion of Medicaid coverage and inquiry into further federal initiatives [3]. National data further showed that 18–44‐year‐old individuals consistently represented ~ 90% of the population that continued to access medical emergency settings for NTDCs, of whom 70% were Medicaid enrollees or uninsured and more likely to be residents of nonurban settings [3]. Notably, in a study sponsored by CMS, Nassah et al. [4] reported that 67% of dentists accepted no Medicaid patients based on low reimbursement rates for service delivery.

Beginning in 2000, Family Health Center of Marshfield (FHC‐M), a regional network of community health centers (CHCs) and other CHCs in Wisconsin have continued to expand the dental safety net by establishing new dental clinics to address critical disparity issues surrounding dental access [5]. Substantial establishment of regional dental access throughout Wisconsin was achieved following the creation of FHC‐M’s 10‐dental clinic infrastructure that serves all patients irrespective of insurance status [5]. Notably, preventive dental care access at FHC‐M dental clinics is available regardless of the patient insurance status or ability to pay. Payer types accepted by FHC‐M dental centers include commercial, Medicaid, uninsured patients with income‐based subsidization based on a sliding fee distribution (SFD) for low‐income populations, and self‐pay. However, administrative data indicated that 10% of patients of FHC‐M dental clinics consistently present with unscheduled dental emergencies despite the mitigation of financial barriers and available access to preventive dental care. Notably, review of FHC‐M data for dental clinic visits across its networked infrastructure during 2018 indicated that >50% of patients who presented at dental clinics with unscheduled dental emergency visits lived within 20 miles of a dental care center with available access to preventive dental care irrespective of insurance status or income. Whereas some patients with NTDCs are referred from medical emergency settings, reasons for unscheduled emergency NTDCs presenting in the dental setting (ED‐NTDC) and delays in seeking preventive care when affordable access for the population is readily available remain unclear.

NTDCs and ED‐NTDCs are generally associated with untreated infectious processes, including periodontal disease (PD) or cavities that progress with painful exacerbations when patients do not seek interventional treatment in dental care settings. Sudden visits with acute dental conditions readily identifiable by Current Dental Terminology (CDT) coding utilized in the dental clinic setting for classifying dental visits negatively impact clinical workflow. In addition, the cost of interventional dental treatment to address the underlying disease is substantially higher compared to the cost of preventive care delivery that could minimize the incidence of ED‐NTDC. Notably, failure to seek timely treatment may result in tooth loss if the affected tooth is irreparably damaged.

The research goal prompted by these observations was to conduct an initial retrospective examination of demographic characteristics abstracted from the electronic health record (EHR) and assess patterns among the cohort of patients between the ages of 18 and 79 years seen between January 1, 2019, and May 19, 2019, residing within a 20‐mile radius of any of the 10 networked FHC‐M dental centers. The clinical cohort was stratified into two subcohorts: those with appointed visits (controls) and those presenting with unscheduled ED‐NTDCs (cases). In Phase II, a prospective survey study was undertaken targeting participants from each subcohort in order to gain more granular insights into behavioral characteristics, dental health literacy, attitudes, and perceived barriers gleaned from survey responses. Defining perceived barriers to dental care access and characterizing behavioral and demographic variables that differ significantly in the subpopulation presenting with unscheduled ED‐NTDC visits are pivotal to informing potential interventional strategies to target reductions in rates of such visits.

## 2. Materials and Methods

### 2.1. Research Approach and Objective

This report summarizes outcomes of an observational, case–control study whose objective was delineation of factors contributing to dental clinic access for unscheduled ED‐NTDCs and demographically profiling patients at the highest risk. A biphasic study design was applied.

### 2.2. Study Design Overview

In Phase I, retrospectively abstracted administrative demographic and clinical characteristics of patients presenting with ED‐NTDCs based on CDT codes detailed below under “population characterization” were compared with patients presenting for routine care at each of the FHC‐M dental centers within the same defined 5.5‐month time interval via electronic query of the integrated medical‐dental record (iEHR) and enterprise data warehouse (EDW) of Marshfield Clinic Health System (MCHS) in order to define each subcohort. Geographic information system (GIS) mapping identified patients who lived within a defined distance from a local dental clinic who met eligibility criteria for further analysis in Phase II.

Phase II applied a mixed‐methods approach to prospective conduct of a survey study to achieve more granular characterization of patient perceptions, behaviors, beliefs, and health literacy that may contribute to the occurrence of unscheduled ED‐NTDCs compared to patients who seek routine preventive dental care. The study sought to identify pivotal factors or gaps in access to service delivery that underlie care‐seeking behaviors associated with unscheduled ED‐NTDC.

### 2.3. IRB Review and Approval of the Study

The study was reviewed and approved by the AAHRPP‐accredited Institutional Review Board of MCHS (IORG#: IORG0000394; FWA00000873), and phase one was exempted from review as all data collection and analyses of clinical and demographic data were initially collected in the context of administrative review with analyses conducted on deidentified, aggregated data sets, including GIS mapping analyses. IRB re‐review was sought for phase two since patient identification for purposes of randomly identifying ED‐NTDC cases to age‐range and gender‐matched controls at each of the dental centers was necessary to mail survey tools to specific patients. The IRB approved return of a completed survey by participants as an indication of voluntary informed consent, as was stated in the cover letter accompanying the mailed survey tool, and use of the participants’ addresses to mail one of four $25 regrets lottery gift cards if their name was randomly selected from among individuals who returned completed surveys.

### 2.4. Population Characterization and Data Collection

In Phase I, interrogation of the iEHR and EDW of MCHS was undertaken to abstract relevant demographic data for the population of patients seeking care at one of 10 networked FHC dental centers between January 1, 2019 and May 19, 2019.

Nonappointed ED‐NTDC cases were identified by CDT codes D0140 and D9110:

• D0140: Limited Oral Evaluation—Problem Focused, which is an examination focused on a specific patient complaint or condition, such as a dental emergency, acute infection, or trauma.
• D9110: Palliative Treatment of Dental Pain, treatment that relieves pain but is not curative; services provided do not have distinct procedure codes.

Patients with scheduled prophylactic dental visits identified by CDT codes D0120, D0150, and D1110 identified controls attending the same dental center as the index cases:

• D0120: “Periodic Oral Evaluation‐Established Patient”, inclusive of evaluation of the entire oral cavity for any existing dental issues, screening for oral cancer, and periodontal screening to evaluate the health of gums and supporting bone structures.
• D0150: “Comprehensive Oral Evaluation‐New or Established Patient”, including medical and dental history review, intraoral and extraoral examination, periodontal assessment, oral cancer screening, and occlusal evaluation.
• D1110: “Routine Dental Cleaning Procedure”, for patients ≥14 years with healthy gums/no PD.

Patients’ access history for NTDC‐related dental or medical care in the 24 months preceding the observational window of this study was further collected in order to characterize the dental access history of cases and controls.

### 2.5. Eligibility Criteria

Eligible cases for inclusion in this case–control study included patients with a documented CDT code, defined above, for an ED‐NTDC visit at one of 10 networked FHC‐M dental clinics within the defined 5.5‐month observational window residing within 20 miles of an FHC‐M dental clinic who were between 18 and 79 years of age.

Eligible controls included adult individuals ages 18–79 years, with scheduled dental prophylaxis evaluations identified by specific CDT codes defined above and were evaluated with cases seen at the same FHC‐M dental clinic within the same temporal window, and requirements for residency within a 20‐mile radius of an FHC‐M dental clinic. Gender was also captured for the controls and cases.

**Figure 1:**
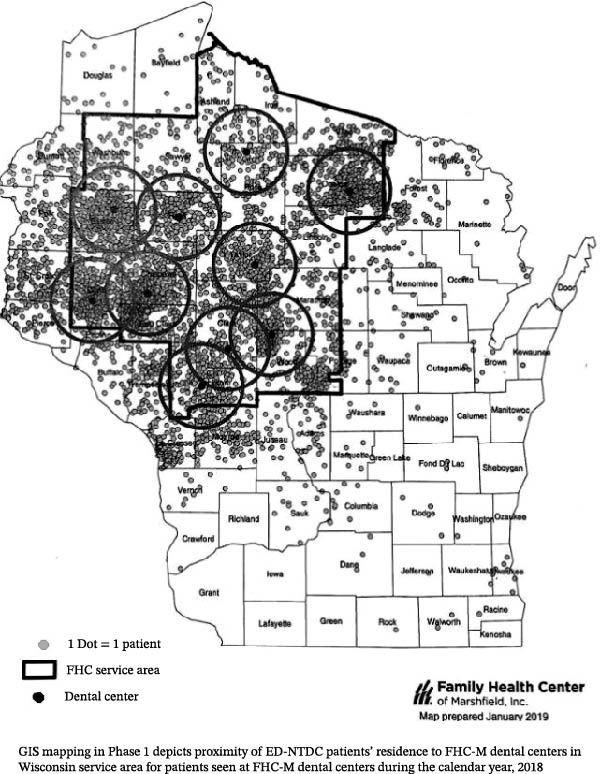
Residence of ED‐NTDC cases relative to FHC‐M dental centers.

The 20‐mile radius was defined by GIS mapping (ArcGis ArcMap, Esri, Redlands, CA) of annual historical data for 2018, and the age range between 18 and 79 years of age (see GIS map, Figure 1). Strategic selection of the 20‐mile radius was informed by a previous study within the same catchment area that explored the impact of the establishment of the FHC‐M dental center infrastructure on rates of NTDCs seen at medical centers pre and postestablishment of the dental infrastructure [2]. The study reported that the highest volume of patients attending dental centers lived in close proximity to these centers, and volume dissipating with longer distance [2]. Moreover, the study reported that nearly 40% of patients with dental emergencies historically lived >10 miles from the medical and dental centers [2]. These observations informed the arbitrary selection of the ≤20‐mile radius criterion used for the current study in the same population. Selecting this eligibility criterion would ensure a high volume of study‐eligible cases regionally where the highest rates of emergency dental visits were documented historically in the previous study [2], while also ensuring availability of matchable controls for each case accessing care at the same dental centers.

Exclusion criteria included trauma‐related ED‐NTDCs, age <18 years or >79 years, and residency exceeding the 20‐mile radius surrounding an FHC‐M dental center defined by GIS analysis.

### 2.6. Statistical Analysis Approach for Phase I

**Figure 2:**
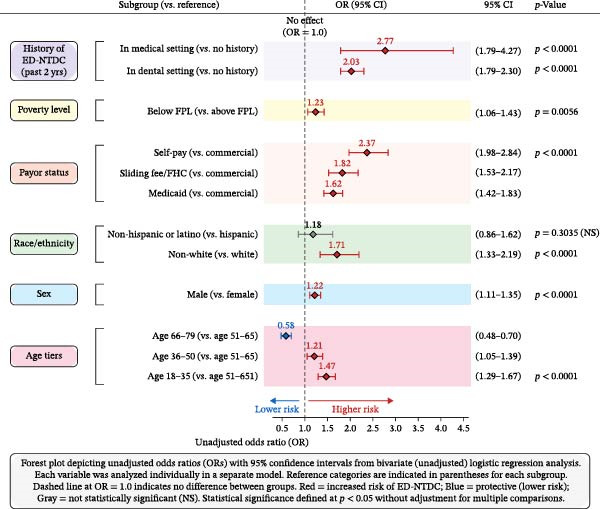
Factors contributing significantly to ED‐NTDC visits at dental clinics following analysis of electronic health records extracted for case and control subcohorts.

Descriptive statistics were applied to deidentified aggregated data sets to summarize characteristics of eligible cases with ED‐NTDC visits (*n* = 1784) and controls (*n* = 18,612) with appointed visits during the observational 5.5‐month window. Bivariate (unadjusted) logistic regression analysis was used to individually model case status (ED‐NTDC = 1, control = 0) as a function of each potential predictor variable [6, 7] (see list of variables as summarized in Table 1). For each variable, one subgroup was designated as the reference category, and unadjusted odds ratios (ORs) with 95% confidence intervals (CIs) were calculated for the remaining subgroups. The overall statistical significance for each variable was assessed using chi‐squared tests. Unadjusted ORs, 95% CIs, and chi‐squared p‐values are summarized in Table 1. Variables achieving statistical significance are further depicted graphically in a forest plot (Figure 2) to facilitate visual comparison of the magnitude and direction of associations across variables.

**Table 1:** Demographic overview of unique patients from Phase I with ED‐NTDC visits at FHC‐M dental centers compared with unique patients attending scheduled dental visits at the same dental center who had no ED‐NTDCs following dental health record review during the defined observational window of the study (1/1/2019–5/19/2019).

Variables	Number of patients with NTDC visits *N =* 1784 (9.3%)	Number of patients without NTDC visits *n =* 18,612 (90.6%)	Odds ratio (OR)	95% CI	Chi sq. *p*‐value
Age					
18–35	743 (41.65)	5541 (31.99)	1.472	1.295–1.673	—
36–50	482 (27.02)	4366 (25.21)	1.212	1.055–1.393	—
51–65	398 (22.31)	4370 (25.23)	Ref	—	<0.0001
66–79	161 (9.02)	3042 (17.56)	0.581	0.481–0.702	—
Gender					
Male	833 (46.69)	7769 (41.74)	1.223	1.109–1.348	—
Female	951 (53.31)	10,843 (58.26)	Ref	—	<0.0001
Race					
Non‐White	75 (5.09)	503 (3.04)	1.710	1.333–2.192	—
White	1398 (94.91)	16,028 (96.96)	Ref	—	<0.0001
Ethnicity					
Non‐Hispanic or Latino	1439 (97.10)	16,113 (96.59)	1.180	0.861–1.616	—
Hispanic or Latino	43 (2.90)	568 (3.41)	Ref	—	0.3035
Payor					
Commercial	338 (18.95)	5349 (28.74)	Ref	—	<0.0001
Medicaid	1006 (56.39)	9870 (53.03)	1.623	1.420–1.833	—
SFD^a^/FHC	226 (12.67)	1962 (10.54)	1.823	1.528–2.174	—
Self‐pay	214 (12.00)	1431 (7.69)	2.367	1.975–2.836	—
Federally defined poverty level status					
Below FPL^b^	226 (12.67)	1962 (10.54)	1.231	1.063–1.426	—
Above FPL^b^	1558 (87.33)	16,650 (89.45)	Ref	—	0.0056
History of NTDC visit within past 2 years at FHC dental center					
Yes	354 (19.84)	2026 (10.89)	2.027	1.788–2.297	—
No	1430 (80.16)	16,586 (89.11)	Ref	—	<0.0001
History of ER visit with NTDC within past 2 years					
Yes	26 (1.46)	99 (0.53)	2.768	1.792–4.274	—
No	1758 (98.54)	18,513 (99.47)	Ref	—	<0.0001

*Note*: ORs presented are unadjusted odds ratios derived from bivariate (unadjusted) logistic regression analysis for each variable individually. FPL status and SFD payor status represent identical patient subsets (226 cases, 1962 controls).

^a^SFD: sliding fee designated (subsidized based on income).

^b^FPL: federally defined poverty level.

Statistical significance was defined at the common 5% level (*p* < 0.05), without adjustment for multiple comparisons.

### 2.7. Phase II: Survey Study Design

#### 2.7.1. Survey Design and Development

**Figure 3:**
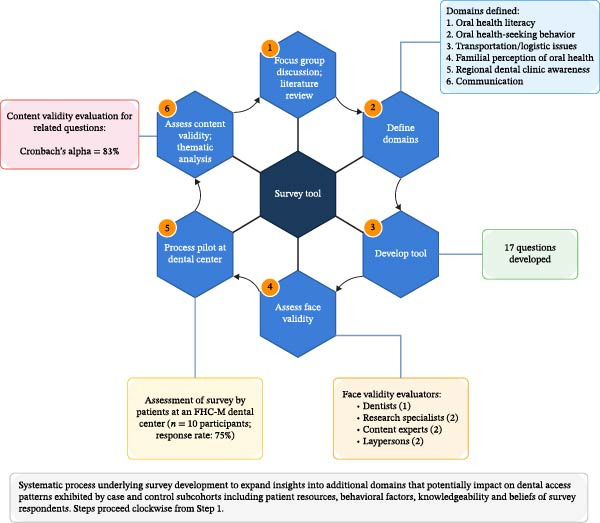
Blueprint for Phase II survey development process.

A literature review revealed the absence of any appropriate validated survey tool for defining variables underlying ED‐NTDC visits to dental clinics, necessitating creation of surveys for cases and controls. A flow chart summarizing steps involved in survey development, validation, and piloting is found in Figure 3. Survey design was informed by a literature review conducted by a dental student intern (Arun Bhatta) and content expert (Ingrid Glurich) versed in survey research. An informal focus group of dental professionals (including dentists, dental hygienists, and patient schedulers) and an interview with the executive director of the FHC‐M dental centers were convened to gain insights into salient points of interest to dental professionals. Foci identified included patterns of care seeking behaviors and patient characteristics in the context of (1) ED‐NTDCs, (2) impact of ED‐NTDC visits on clinical workflow, and (3) scheduling of patients for further follow‐up. The literature review further defined six domains for inclusion in the survey tool as shown in Figure 3, with refinements informed by feedback from dental professionals. Thematic analysis by two research specialists (Neel Shimpi and Ingrid Glurich), who were content experts with training in survey design was further conducted on responses to open‐ended questions captured during beta testing of the survey tools. The survey was designed for a 5^th^ grade reading level. Final versions of the case and control survey tool are available in Supporting Information 1: Appendix C1 and Supporting Information 2: Appendix C2, respectively. The license for these survey tools is held by the authors, and full permissions for free use are granted for other entities without requirement for further permissions when the source of the tools is appropriately cited.

### 2.8. Validity Assessment and Beta Testing

Survey content validity assessment included face validity determination by (1) content experts and dental clinicians, (2) experts in survey design, and (3) lay individuals, to ensure relevance, clarity, and comprehensibility of the questions, respectively. The survey was further beta‐tested by 10 volunteers at an FHC‐M dental clinic who anonymously evaluated the contents for comprehension, clarity, and sensitivity of the survey questions. Finally, construct validity was determined to measure the internal consistency of two or more questions examining the same endpoint to determine correlation between responses by participants for these questions.

### 2.9. Survey Distribution

Prospective survey mailings were distributed in phases applying the process defined by Chyou et al. [8] for the distribution of dental surveys. Briefly, achievement of the targeted survey response rate is supported by monitoring response rates to mailing the surveys in 3 phases and projecting the number of surveys targeting potential cases and controls for inclusion in consecutive mailings to achieve the targeted response rate based on the survey response rate for each consecutive mailing [8].

### 2.10. Statistical Analysis

Inter‐rater reliability for thematic analysis was calculated with Cohen’s kappa statistic. The construct internal consistency was determined by calculating the Cronbach alpha statistic. Calculations resulted in the distribution of 1600 surveys to ED‐NTDC cases and achievement of a 5% response (*n* = 84 completed surveys) and 2700 surveys to controls with achievement of a 4% response rate (*n* = 112 completed surveys).

Descriptive statistics compared the responses of cases and controls to survey questions. These analyses involved definition of the number of participants among cases or controls who selected a specific potential response from among the possible choices (numerator) over the total number of cases or controls who selected from among any of the potential responses for the specific question, expressed as a percentage. Analytical results shown in Supporting Information 3: Appendix Table B. Questions where cases and controls showed divergent percentages in their response rates were flagged as potential additional risk factors for ED‐NTDC, which were not identifiable by medical record interrogation because these represented patient‐specific beliefs, habits, knowledge, reflected family history, or characteristics that were identifiable by analysis of the survey tool responses not captured in the EHR.

## 3. Results

### 3.1. Phase I Results

Table 1 summarizes results of the iEHR review and presents the demographic profiles of patients classified as ED‐NTDC cases or patients attending scheduled nonemergency dental visits (controls) who accessed periodic assessment and preventive dental care at any of the 10 FHC‐M dental clinics between January 1, 2019 and May 19, 2019, and met geographic study eligibility criteria. ED‐NTDC cases accounted for 9.3% of the unique patients seen during the observational temporal window. Supporting Information 3: Appendix Figure A depicts the percentage of ED‐NTDC cases among all patients seeking dental access across each of the 10 operative regional dental centers during the defined temporal window. Bivariate (unadjusted) logistic regression analysis identified statistically significant associations between ED‐NTDC case status and the following variables: age (*p* < 0.0001), sex (*p* < 0.0001), race (*p* < 0.0001), payor status (*p* < 0.0001), FPL status (*p* = 0.0056), history of prior ED‐NTDC at an FHC‐M dental center (*p* < 0.0001), and history of ER visit with NTDC (*p* < 0.0001). Ethnicity was not statistically significant (*p* = 0.3035). The forest plot (Figure 2) provides a graphical depiction of the unadjusted ORs for variables achieving statistical significance, facilitating visual comparison of the magnitude of association for each variable relative to its reference subgroup.

### 3.2. Phase II Results

The construct internal consistency of survey tools achieved 83%. Inter‐rater reliability for thematic analysis achieved 89%. The 2‐week process pilot conducted at one FHC‐M dental clinic to beta test the survey tools had a response rate of 75%. The final versions of the Case and Control Survey tools distributed by mail to survey study participants in Phase II are found in Supporting Information 1: Appendix C1 and Supporting Information 2: Appendix C2, respectively. Prior to distribution, the survey was anonymously beta tested by 10 patients attending a dental visit at an FHC‐M dental center who volunteered to complete and assess the survey by completing the survey instrument assessment questionnaire. The participants rated survey questions as clear, easy to understand, and acceptable (see survey instrument assessment questionnaire and summary of participant scoring, Supporting Information 3: Appendix C3).

Survey distribution achieved the targeted 4% response rate both among case and control respondents presenting at the same FHC‐M dental center among the 10 FHC‐M Dental Centers during the defined study window. Among the total number of surveys targeted to cases distributed in three separate mailings (*n* = 1600), 84 were completed and returned. Similarly, among the total number of surveys targeting patients attending scheduled prophylactic dental visits (control population, *n* = 2700), 112 completed surveys were returned. Distribution of higher numbers of surveys was required among patients attending scheduled dental visits to achieve the targeted response rate due to lower initial rates of completed survey returns in the first 2 waves of survey distribution.

Table 2 delineates reasons for ED‐NTDC visits based on the provision of additional feedback volunteered by a subset of patients. Approximately 12% of ED‐NTDC visits in the dental setting were related to unpreventable emergent conditions. In‐depth comparisons of survey responses by all case and control respondents are summarized in Supporting Information 3: Appendix Table B.

**Table 2:** Case survey respondents’ opinions on dental access at FHC‐M dental centers.

Respondent‐reported opinions re: dental center operations/services	# (%)
I was aware of FHC dental centers prior to ED‐NTDC visit	65/82 (79%)
Making/finding convenient appointments is difficult	8/82 (10%)
FHC dental appointments work with my schedule	46/82 (56%)
Dental personnel emphasize importance of tooth/gum care	40/82 (49%)
I can get care at FHC dental centers regardless of ability to pay	23/82 (28%)
FHC dental centers see patient regardless of Insurance status	26/82 (32%)
I believe that dental care is not affordable at FHC dental centers	6/82 (7%)
FHC dental centers do not treat my type of dental issues	6/82 (7%)

Self‐reported contributory factors surrounding ED‐NTDC visit (*n* = 57 respondents)	# (%)
I only make dental appointments if needed	16/57 (28%)
I could not get an appointment when needed	6/57 (10.5%)
The ED‐NTDC was due to an emergent condition	10/57 (17.5%)
I could not afford earlier preventive dental appointments	11/57 (19%)
I had no dental insurance	10/57 (17.5%)
Work schedule prevented being seen at the dental clinic	1/57 (2%)
I have a fear of dental visits	3/57 (5%)

As depicted in Supporting Information 3: Appendix Table B, substantially higher survey response rates among case and control participants were noted in the 65–79‐year‐old age tier and lower rates in the 18–44‐year‐old age tier, and respondents were over 60% female. Further noteworthy observations in Supporting Information 3: Appendix Table B are as follows. The percentage of case and control participants, respectively, with no commercial insurance coverage decreased with age, with 83% and 74% of 18–44‐year‐old, 74% and 75% of 45–64‐year‐old, and 51% and 39% of 65–79‐year‐old, respectively, having no commercial insurance. Other notable differences included less formal education among case vs. control participants and higher response rates from female vs. male respondents. Similar distributions for racial and ethnic background and employment status were noted among the subcohorts.

Salient differences in responses by case and control participants were noted in response to questions related to the importance of dental access, family history of dental access, payor status, and transportation status, as shown in Supporting Information 3: Appendix Table B. In the context of thematic domains shown in Figure 3 and oral health‐seeking behaviors, case vs. control respondents were more likely to have a painful tooth pulled rather than saved (14% vs. 2%) and exhibited delay in seeking dental care in the presence of dental pain (22% vs. 14%). Differences between cases and controls were noted in perceptions and practice surrounding annual frequency of dental visits with lower percentage of cases vs. controls reporting twice annual dental visits (34% vs. 61%, respectively, and higher percentage of cases vs. controls reporting “no annual dental visit or only ‘as‐needed’ access,” 37% vs. 3%, respectively). In the context of oral health literacy, cases more frequently agreed that dental visits were only necessary for dental emergencies (16% vs. 2%). Case participants also reported higher rates of dental fear and anxiety surrounding dental visits compared to control counterparts (19.5% vs. 11%). In the context of the theme: familial perception regarding the importance of oral health, case participants were less likely to have had regular dental visits as a child (43% vs. 68%). Further, case participants reported higher rates of early tooth loss of a parent compared to control participants (32% vs. 18%, respectively). In the context of transportation and logistical issues surrounding dental access, differences in the number of case participants reporting medical support vehicle utilization (7% vs. 3%), or lack of transportation were noted compared to control participants (11% vs. 3%). In the context of knowledge‐based themes, differences between cases and controls were noted in perceptions and practice surrounding frequency of dental hygiene and annual frequency of dental visits with lower percentages of case vs. control participants reporting tooth brushing twice daily (43% vs. 51%, respectively) and twice annual dental visits or no annual dental visit or only “as‐needed” (37% vs. 3%, respectively). With respect to communication thematic analysis, cases reported high awareness of FHC‐M dental centers (79%), and 56% reported compatibility of dental appointment availability with their schedules.

## 4. Discussion

The study identified variables reflecting statistically significant differences between case and control subcohorts based on unadjusted ORs derived from bivariate (unadjusted) logistic regression analysis, as summarized in Table 1 and depicted graphically in Figure 2. In Phase I, ~9% of dental visits seen across the 10 FHC‐M regional dental centers during the ~ 5.5‐month study window were identified as ED‐NTDC visits from patients living within a 20‐mile radius of an FHC‐M dental center. This rate is lower than the 20% rate reported for adults by Fiehn et al. [9], who modeled dental claims data from 13 US states captured between 2013 and 2017 in the IBM Watson Medicaid Marketscan database for emergency dental encounters identified by CDT codes among enrollees. The current study found that compared to patients with commercial payor status, those with “self‐pay” status had the highest OR of presenting with a dental emergency, followed by FHC‐M’s subsidized “sliding fee” (SFD/CHC) payor status, which is determined based on income and Medicaid payor status. Patients falling into the lower federally defined poverty level (FPL), non‐White, or male also exhibited higher ORs for ED‐NTDC visits versus corresponding comparator subgroups. Patients aged 18–35 years had a higher OR (1.4) than other age groups for presenting with ED‐NTDCs. A recent Health Policy Institute (HPI) report (2023) showed similar trends, citing the lowest rates of dental visits among uninsured adults aged 19–64 years in 2022 [10]. This age group also had the highest rates of low dental access among those in <100% FPL (20%) and 100%–200% FPL (25%), compared with ≥300% FPL groups where public health programs may offer financial assistance [10]. Cost was also a barrier to needed dental care access, far exceeding the cost as a barrier to other healthcare access in the 19–64‐year‐old age group. Non‐White race was also associated with lower dental access in the HPI report [10].

To amplify insights into factors underlying patient behaviors, survey instruments were created to identify variables contributing to ED‐NTDC and were targeted to the cohort characterized in Phase I. Domains selected for inclusion in survey design included social determinants of health (SDOH), including patients’ demographics, health literacy, perceptions/knowledge regarding their eligibility for dental care access, beliefs or behaviors influencing how they access dental care, personal and familial history surrounding dental care access and attitude towards dental hygiene, importance of dental health, and compliance with preventive practices that promote oral health.

Availability of the newly developed, validated survey tools is expected to support determination of prevalence of factors that define negative and positive dental care access behavior within defined populations in disparate geographic settings to assist stakeholders within the public health domain in identifying persistent, unresolved, or perceived barriers reported by the subpopulation that access dental clinics with ED‐NTDCs. Survey responses delineated differences between case and control cohorts within the FHC‐M dental center service areas, with most noteworthy observations summarized previously in “Results.” Among cases (57/82 [70%]) who offered reasons contributing to their ED‐NTDC visit, leading reasons included making visits only as needed (28%), financial barriers for preventive care (19%), lack of dental insurance (18%), emergent condition (18%), and lack of appointment availability when needed (11%).

Compared to the analysis of costs associated with NTDC visits in the medical setting, analyses of cost implications surrounding ED‐NTDC visits are not extant. While a 2024 American Dental Association report estimated an average cost of $749 per emergency treatment in the medical setting, totaling to an annual nationwide cost totaling ~$1.6 billion annually with 33% covered by Medicaid, the estimated average national cost per preventive dental visit was between $180 and $211 and average cost per visit for restorative dental care between $300 and $592 [11]. Recent analysis of Cigna [12] data from 2018 found that clients lacking a history of consecutive annual preventive dental visits had a 1.5‐fold increased likelihood of developing PD compared to clients attending such visits. The report also noted that annual medical cost savings were realized in the year following PD treatment initiation at a cost of ~$759 (or ~ 10%) annually per member (PMPY) [12]. Moreover, Cigna clients who complied with periodontal maintenance compared to those who did not had ~ 22% fewer dental emergency visits [12]. For enrollees with diabetes, which is exacerbated by inflammatory processes related to PD, medical cost savings achieved ~ 18% in the year following 3 years of periodontal maintenance and totaled 12.25% medical cost savings over 5 years in diabetics compared with 4.5% in subjects not complying with annual prophylaxis [12]. Following interrogation of the IBM Watson Marketscan Medicaid database, Okunev et al. [13] similarly reported that cost for Medicaid enrollees attending annual preventive care from 2015 to 2019 was 43% lower than those receiving no dental prophylaxis, and that those with no preventive care had an 8‐fold increased likelihood to present with an NTDC, 7‐fold increase for oral surgery risk, and 6‐fold increase in requirement for dental‐related opioid prescription.

Extensive dental coverage under Medicaid is offered by only 18 (36%) of US states, while 16 and 11 states, respectively, offer limited or emergency only dental coverage, and 5 states currently offer no coverage [14]. Notably, reimbursement policies, no‐ or insufficient‐dental coverage through Medicare and Medicaid, and disparity populations who cannot afford dental insurance or out‐of‐pocket cost contribute to sustained prevalence of ED‐NTDC visits, presenting an unsolved issue for stakeholders [14]. Infrastructural support for medical care access is largely lacking in the context of dental access, contributing to multifactorial access barriers, discrepant dental access behaviors, and sustainment of disparity populations facing significant reductions in quality of life and overall health consequential to untreated poor oral health [15]. In Phase I analyses of the current study, economic factors emerged most strongly as contributors to ED‐NTDC visits and underline a compelling need for implementing reforms to current oral health access financial infrastructural supports.

The following study limitations are acknowledged. During Phase I, data surrounding patient access to dental services outside a CHC‐dental infrastructure may not have been captured during the time periods examined in the study.

Study limitations to be acknowledged for Phase II include variable response rates among cases/controls respondents with the lowest response in the age group 18–35 years and disproportionately higher response among the 65–79‐year‐old age group for the survey study component. However, in Phase I, where interrogation of the iEHR supported analysis of all patients, these differences reflected that numbers of patients accessing care at dental centers for both ED‐NTDCs and prophylactic care were highest in the 18–35‐year‐old age group compared with the 65–79‐year‐old age group. Reasons for lack of participation by the younger age group in the survey study are unknown but limited the meaningful conduct of more rigorous statistical analysis beyond descriptive analysis of the survey outcomes reported. Finally, variability in denominator values noted in tables summarizing survey outcomes data further indicated that some participants did not provide a response to every survey question. Additionally, in the present study, Phase I analyses were mainly conducted using bivariate (unadjusted) logistic regression analysis for each variable individually; accordingly, the reported unadjusted ORs do not account for potential confounding between variables. Future studies incorporating multivariate logistic regression analysis to estimate adjusted ORs would further clarify which variables independently predict the ED‐NTDC risk after controlling for other covariates. Such analyses will be more informative following expanded collection of additional survey data targeting achievement of more balanced response rates from participants that have been subset to tiers in order to independently predict ED‐NTDC risk, controlling for all potential covariates, including candidate risk factors identified in Phase II, that contribute to risk and achieve significance levels that merit their inclusion in multivariate logistic regression models.

Rates of ED‐NTDC for the remainder of 2019 were also examined for the poststudy temporal window to examine the potential for seasonal variability. ED‐NTDC visits during the study window comprised 34% of all annual ED‐NTDC visits to the FHC‐M dental clinic enterprise. Notably, increases in ED‐NTDC rates were observed in summer and fall months across FHC‐M dental centers (Supporting Information 3: Table D). Several potential factors are posited to contribute to seasonal variability, including potential travel avoidance due to winter weather conditions which coincided with the study’s observational window. Additionally, an influx of migrant populations occurs from late spring through fall to aid with agricultural work occurring in the largely rural areas surrounding dental center locations, and this transient subpopulation may expand the monthly rate of patients seeking treatment for emergent ED‐NTDCs at FHC‐M dental centers during the poststudy temporal window. These findings may have implications for dental center staffing in accommodating patient care delivery in the context of challenges to workflow caused by care delivery to unscheduled patients and the volume of patients treated based on availability of professional staff. To examine this, an additional analysis was performed to assess the impact of clinical practice days at each center by month during calendar year 2019 with respect to the number of ED‐NTDC patients with valid emergency CDT codes treated by comparing the 5‐month study window with the 7‐month post‐ study window. Data summarized in Supporting Information 3: Table E indicate that expansion in staffing noted at FHC‐M dental centers between June–December 2019 correlated with increased capacity to treat ED‐NTDC patients across all FHC‐M dental centers. Taken together, factors underlying the perceived “seasonal variability” in ED‐NTDC access are potentially multifactorial. Nonetheless, the population presenting with dental emergencies during the time frame examined in the current study is representative since historically, the regional population has been shown to be very stable over time as borne out by the overlap of the FHC‐M service area with that of the Marshfield Epidemiological Study Area (MESA), whose population is tracked annually to support the conduct of epidemiological research [16]. Moreover, for noncommercially insured and low‐income patients who comprise a majority of the patients seen at FHC‐M dental centers, seeking care at FHC‐M dental centers is currently their only viable option for clinical access for ED‐NTDC management. Insufficient reimbursement stipulated by governmental policies for patients on Medicaid caused the majority of regional dental providers to decline the provision of care to patients on Medicaid.

## 5. Conclusions and Implications for Practice and Policy

As summarized above, distinct clinical, demographic, and behavioral factors, including SDOH, were noted for patients who sought emergency dental access at local FHC‐M dental clinics comprising a dental safety net infrastructure compared to patients with comparable geographic proximity who chose prophylactic care access at the same facility during the same temporal window. The current study developed and utilized novel survey tools to assess patients’ perspectives, beliefs, health literacy, clinical experiences, and demographic factors that may influence patients’ dental access behaviors. The public availability of these new survey tools addresses a gap in the oral health literature for determination of key factors that govern patients’ dental access behaviors with adaptability for use in dental clinics in discrete geographic settings. An improved understanding of patient perspectives informs opportunities for patient education and interventional remediation to achieve more appropriate and cost‐effective dental care access, especially for populations consistently citing economic or other barriers to dental care access. Finally, persistent and additional gaps highlighted by study findings reveal a need for further health policy reforms, especially in the dental public health sector, to improve appropriate cost‐effective dental access.

## Author Contributions

In compliance with ICMJE criteria, Ingrid Glurich, Aloksagar Panny, Neel Shimpi, Mary Dorsch, Arun Bhatta, Richard Berg, Amit Acharya, and Gregory Nycz contributed substantially to the conception and design of, or acquisition of data or analysis and interpretation of data, and drafting the article or revising it critically for important intellectual content. Individual roles of authors are fully delineated in the CRediT classification document requested by the Journal Editor.

## Funding

Institutional support was received from Family Health Center of Marshfield, Inc. and the Marshfield Clinic Research Institute.

## Disclosure

This article does not report on a clinical trial and no preregistration as an independent institutional registry surrounding study design, variables, treatment conditions, analyses, or statistical modeling has been filed. The authors grant full permissions for free use of the survey tools found in Appendix C when the source from which the tools were obtained is fully cited. All the authors have contributed to the final approval of the version to be published. This study was undertaken to further clarify results emanating from the administrative review of FHC‐M clinical data to inform service delivery improvement by defining subpopulations still experiencing barriers to dental care access, wherein clinic support was utilized for administrative data review and follow‐up. The executive directors participated as study authors as delineated in the CRediT classification document and contributed institutional support for the costs of the administrative study, which represents a collaborative effort of MCRI scientists and FHC staff who are employees of FHC‐M and MCRI. Use of survey tools by other researchers is associated with no cost, so there is no commercial benefit to the authors in association with this study.

## Ethics Statement

The study was reviewed and approved by the AAHRPP‐accredited Institutional Review Board of Marshfield Clinic Health System (IORG#: IORG0000394; FWA00000873), and all work with human subjects was done in accordance with the Declaration of Helsinki. The manuscript was prepared using the STROBE guidelines checklist, which has been submitted to the editor.

## Consent

Survey participants were informed that voluntary completion and return of the study survey constituted informed consent approval, and the cover letter and survey tools were reviewed and approved by the IRB.

## Conflicts of Interest

Notably, during the tenure of the study, two authors, Dr. Amit Acharya was serving as the Executive Director of MCRI and Gregory Nycz was (and continues in the role of) Chief Executive Officer of FHC‐M. No conflicts of interest are declared relative to their administrative duties, for their institutions, or for other co‐authors participating in the study.

## Supporting Information

Additional supporting information can be found online in the Supporting Information section.

## Supporting information


**Supporting Information 1** Appendix C1: The final survey tool distributed to ED‐NTDC cases presenting at FHC‐M dental centers during the study window from January 1,2019 through May 19, 2019, is shown.

**Supporting Information 2** Appendix C2: The final survey tool distributed to age‐range, gender‐matched, and FHC‐M dental center‐matched controls attending a scheduled during the study’s temporal window from January 1,2019 through May 19, 2019, is shown.

**Supporting Information 3** Appendix Table B: Table reports the aggregate data summarizing case and control responses selected for each individual survey question included in the case and control survey tools (Appendix C1 & C2, respectively). Data shown summarizes the number of respondents selecting each of the possible choices for any given question divided by the number of participants who answered the question and percentage of respondents that selected each specific potential response to each of the survey questions. Comparison between frequency of response selection by case and control survey participants for each corresponding question are shown. Appendix Figure A: Figure depicts the prevalence per 100,000 of ED‐NTDC visits across each of the 10 regional FHC‐M dental centers within the context of the total number of patients who logged dental visits at each of the 10 dental centers during the study window from January 1, 2019 through May19, 2019. Appendix C3: The survey tool assessment questionnaire is shown. The questionnaire was completed by 10 anonymous volunteers during beta testing of the case/control surveys following a dental visit at an FHC‐M dental center in Marshfield WI. Volunteers were requested to complete the survey instrument followed by completion of the survey assessment questionnaire to beta test acceptability and comprehensibility of the survey tools by patients. Responses of the volunteer participants is summarized for each of the evaluations that were requested. Table D: Table depicting annual utilization patterns surrounding access for ED‐NTDCs in 2019 based on CDT coding at each of the dental clinics. The study’s observational window (spanning January 1, 2019 through May 19, 2019), is presented along with ED‐NTDC patient counts for the post‐observational period from May 20, 2019 through December 31,2019 and provides evidence of potential seasonal variability. Table E: Depicts the ratio of ED‐NTDC cases seen at each FHC‐M dental center during the ~5‐month observational window of the study and the ~7 post‐study months of 2019 juxtaposed to clinical practice days (CPD) logged monthly at each of the dental centers throughout the year. Ratios indicate a correlation between increased CPD and increased capacity to treat ED‐NTDC cases in the post‐study window. These data support high rates of ED‐NTDC presenting to FHC‐M service area and the high need for services to a population with high dental disparity access.

## Data Availability

The data can be available using the “Editor instead” option.
