# Supplementary material for: Delineating Factors Surrounding Emergency Dental Access Behavior for Nontraumatic Dental Conditions Among Patients With Available Access to Local Preventive Dental Care
Source: Int J Dent. 2026 Jun 24;2026:9509544. doi: 10.1155/ijod/9509544 (PMC13291887; doi:10.1155/ijod/9509544)
Supplement: Supplementary file 1 — Supporting Information 1 Appendix C1: The final survey tool distributed to ED‐NTDC cases presenting at FHC‐M dental centers during the study window from January 1,2019 through May 19, 2019, is shown. [file IJOD-2026-9509544-s001.docx]

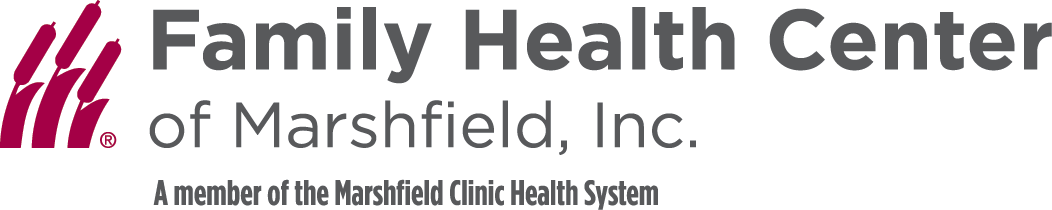

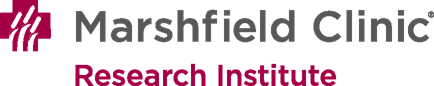


*Supporting Information 1*

*Appendix C1: Case*

Survey: Oral Health

Your answers will help us to improve the information and care that we provide to our patients related to oral/dental health. This survey is voluntary and will take about **5-7 minutes** to finish.

1. **What is the highest grade you completed in school?**

Less than high school

High school graduate

Some college or associate degree

4-year college graduate

More than 4-year college degree (professional)

1. **Employment:**

Employed for wage or salary

Self-employed

Out of work for less than 1 year

Out of work for 1 year or more

Taking care of house and family

Going to school

Retired

Unable to work for health reasons or disability

1. **Generally, how often do you brush? (select one)** Once a day

Twice a day

More than twice a day

I do not brush daily

1. **Dentists recommend that a person should brush his/her teeth: (select one)**

One a day

Twice a day

After each meal

Only brush when food gets stuck in teeth

1. **What reason kept you from making an earlier dental appointment that might have prevented your dental emergency?**

__________________________________________

__________________________________________

__________________________________________

1. **How often do you make a regular dental visit?**

Once a year  Twice a year

More than twice a year

As needed if I have dental problem

I don’t make regular dental visits because:

__________________________________________

__________________________________________

1. **What is the recommendation for how often a person should visit a dentist?**

Once a year

Twice a year

More than twice a year

As needed if there is a dental problem

There is no guideline for how often a person

should visit a dentist

1. **Oral diseases can contribute to making chronic health conditions such as diabetes, heart disease or chronic kidney disease worse**

True

False

I don’t know

1. **Are the following statements true or false?**

|  | True | False | I don’t know |
| --- | --- | --- | --- |
| It is important to brush your teeth at least once a day |  |  |  |
| Regular dental visits can prevent both oral pain and expensive dental treatment |  |  |  |
| Some infections in the mouth can be life threatening |  |  |  |
| Visiting a dentist is only needed if your mouth is unhealthy |  |  |  |
| Dental problems transfer across generations and dentists cannot prevent them |  |  |  |

1. **Have any of the reasons listed below ever kepy you from seeing a dentist (check all that apply)**

I could not get a dental appointment when needed

I could not afford dental care

I did not have dental insurance coverage

I did not have transportation

Dental provider was not handicapped accessible

I am afraid of seeing a dentist

Dental office did not accept my insurance

Dentist did not accept Medicaid patients

Other (please specify): _____________________

___________________________________________

**TURN**

1. **If you are experiencing a lot of gum or tooth pain, which of the following are you most likely to do? (select one)**

Take pain medication and make a dental

appointment

Go to the hospital Emergency Department

Take some pain medication, wait and see if

problem goes away

Make an emergency dental visit

Other (please specify): _____________________

___________________________________________

1. **If you have to go to a dentist tomorrow, how would you feel about it? I would: (Select one)**

Expect it to be a reasonably enjoyable experience

Not care one way or the other

Be little uneasy about it

Be afraid that it might be unpleasant or painful

Be very frightened of what the dentist would do

Rather skip the dental appointment

1. **Which of the following statements do you agree with? (check all that apply)**

My teeth are going to fall out anyway, saving them

is not worthwhile

I would only visit a dentist when pills don’t control

my dental pain

I would rather have the tooth pulled than save it

I only need to seek dental care if I have a dental

emergency

I think it is important to see a dentist at least once

a year

1. **My parents/caregiver: (Select all that apply)**

Only visited dentists if they had dental problems

Did not like going to dentists

Took me to see the dentist at least once a year

Encouraged me to brush my teeth regularly

Lost multiple teeth by the age 50 years

1. **How did you get to the dental clinic for your latest dental appointment (select one)**

Bus

Family/own vehicle

Medical Transport Management (MTM) vehicles

Family/own vehicle

Medical Transport Management (MTM) vehicles

Got a ride from neighbor/friend/family member

Other (please specify):_____________________

__________________________________________

1. **Do you have a car or driver available to bring you to a dental appointment when needed?**

Yes

No

Sometimes

1. **Before your dental emergency visit at the FHC dental center you attended/or you are currently attending, were you aware of this dental center?**

Yes

No

I don’t know

***If you checked ‘No’ or ‘I don’t know’ to question 17, please go to Q 19.***

1. **Please select all that apply: Regarding your past experience with Family Health Center (FHC) Dental Center:**

I have/had problems receiving dental care when I

needed it

I know that it provides dental services whether

you can afford it or not

I know that it provides dental services regardless

of your insurance status

Dental treatment is not affordable there

Dentist do not treat the main dental problem that I

have

Dentist and staff do not treat me with respect

I have had a hard time making an appointment

there

Dentist and staff advise me on how to keep my

teeth and gums healthy

Dental center staff help me make appointments

that work with my schedule

1. **Is there something the Family Health Center-Dental Center could do that would increase your willingness to make an appointment for a dental checkup?**

____________________________________________________________________________________________________________________________________________________________________________

***Enter me in the lottery to win a $25 gift card***

***Yes No thanks***
