# Supplementary material for: Delineating Factors Surrounding Emergency Dental Access Behavior for Nontraumatic Dental Conditions Among Patients With Available Access to Local Preventive Dental Care
Source: Int J Dent. 2026 Jun 24;2026:9509544. doi: 10.1155/ijod/9509544 (PMC13291887; doi:10.1155/ijod/9509544)
Supplement: Supplementary file 3 — Supporting Information 3 Appendix Table B: Table reports the aggregate data summarizing case and control responses selected for each individual survey question included in the case and control survey tools (Appendix C1 & C2, respectively). Data shown summarizes the number of respondents selecting each of the possible choices for any given question divided by the number of participants who answered the question and percentage of respondents that selected each specific potential response to each of the survey questions. Comparison between frequency of response selection by case and control survey participants for each corresponding question are shown. Appendix Figure A: Figure depicts the prevalence per 100,000 of ED‐NTDC visits across each of the 10 regional FHC‐M dental centers within the context of the total number of patients who logged dental visits at each of the 10 dental centers during the study window from January 1, 2019 through May19, 2019. Appendix C3: The survey tool assessment questionnaire is shown. The questionnaire was completed by 10 anonymous volunteers during beta testing of the case/control surveys following a dental visit at an FHC‐M dental center in Marshfield WI. Volunteers were requested to complete the survey instrument followed by completion of the survey assessment questionnaire to beta test acceptability and comprehensibility of the survey tools by patients. Responses of the volunteer participants is summarized for each of the evaluations that were requested. Table D: Table depicting annual utilization patterns surrounding access for ED‐NTDCs in 2019 based on CDT coding at each of the dental clinics. The study’s observational window (spanning January 1, 2019 through May 19, 2019), is presented along with ED‐NTDC patient counts for the post‐observational period from May 20, 2019 through December 31,2019 and provides evidence of potential seasonal variability. Table E: Depicts the ratio of ED‐NTDC cases seen at each FHC‐M dental center durin [file IJOD-2026-9509544-s003.docx]

**Supporting Information 3: Appendix Figure A**:

Figure depicts the prevalence per 100,000 of ED-NTDC visits across each of the 10 regional FHC-M dental centers within the context of the total number of patients who logged dental visits at each of the 10 dental centers during the study window from January 1, 2019, through May19, 2019.


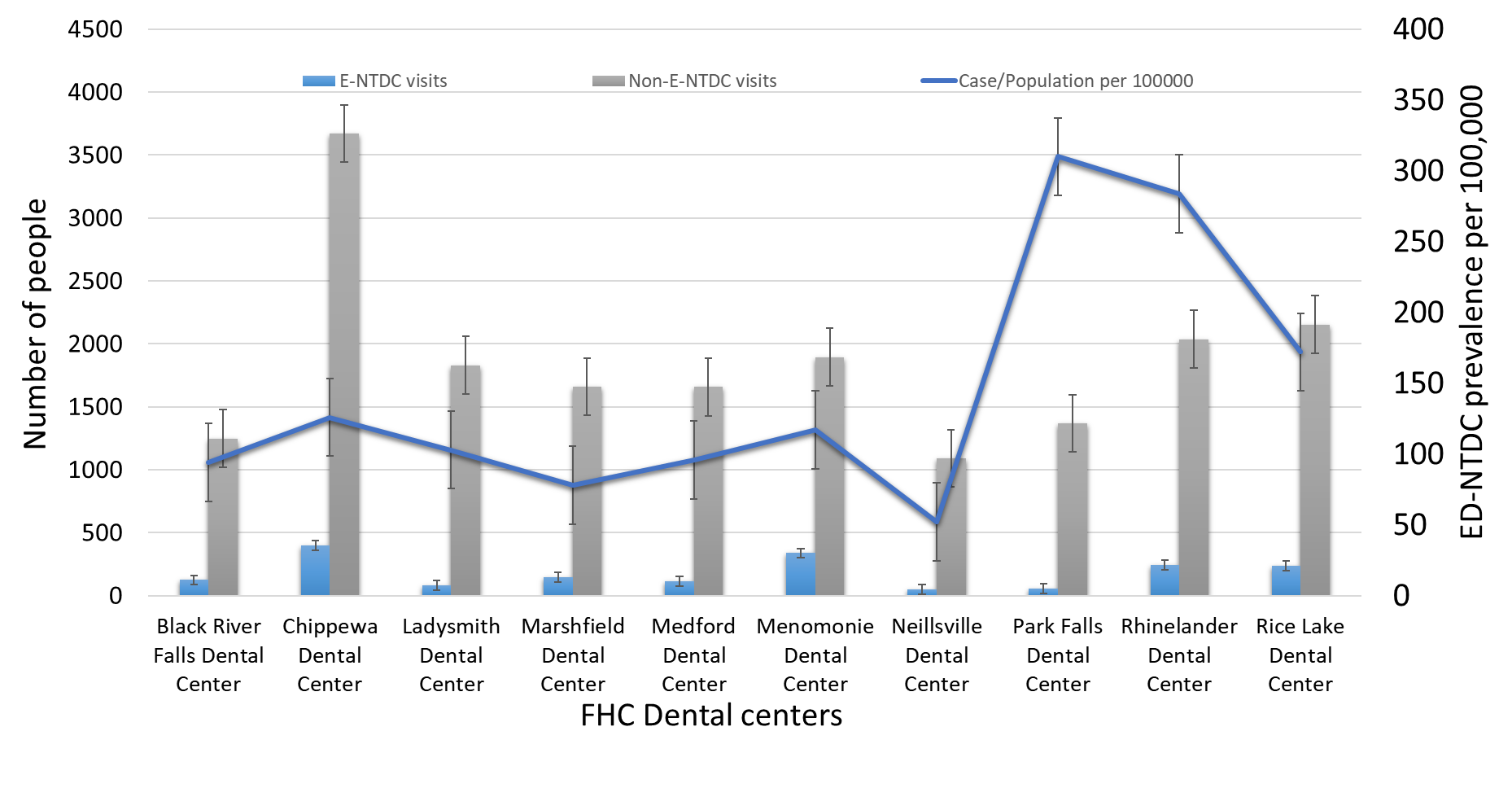


| Supporting Information 3 Appendix Table B: Table reports the aggregate data summarizing case and control responses selected for each individual survey question included in the case and control survey tools (Appendix C1 & C2, respectively). Data shown summarizes the number of respondents selecting each of the possible choices for any given question divided by the number of participants who answered the question and percentage of respondents that selected each specific potential response to each of the survey questions. Comparison between frequency of response selection by case and control survey participants for each corresponding question are shown. | | |
| --- | --- | --- |
| *Appendix Table B: Summary of Survey Responses* | | |
| Demographic Overview | ***Cases*** | ***Controls*** |
| *Educational profile of respondents* |  |  |
| < High School/High School Graduate | 43/82 (52%) | 44/111 (40%) |
| Some college/Associates /4 year/ or professional degree | 39/82 (48%) | 66/111 (60%) |
| *Employment status of respondents* |  |  |
| Employed (salary or wage) | 18/84 (22%) | 21/113 (19%) |
| Self-employed | 6/84 (7%) | 13/113 (11.5%) |
| Unemployed | 2/84 (2%) | 4/113 (3.5%) |
| Taking care of home/family | 4/84 (5%) | 4/113 (3.5%) |
| Student/Volunteer | 1/84 (1%) | 1/113 (1%) |
| Retired | 36/84 (43%) | 50/113 (44%) |
| Unable to work due to health status/disability status | 17/84 (20%) | 20/113 (18%) |
| *Age distribution of respondents* |  |  |
| 18-44 years | 6/84 (7%) | 23/113 (20%) |
| 45-64 years | 35/84 (42%) | 40/113 (35%) |
| 65-79 years | 43/84 (51%) | 50/113 (45%) |
| *Gender of Respondents* |  |  |
| Male | 34/84 (40%) | 37/113 (33%) |
| Female | 50/84 (60%) | 76/113 (67%) |
|  |  |  |
| *Racial identification by respondents* |  |  |
| Asian | 1/84 (1%) | 0 |
| Black | 0 | 0 |
| White | 73/84 (87%) | 100/113 (88.5%) |
| Multi-racial | 2/84 (2%) | 0 |
| Declined | 8/84 (10%) | 13/113 (11.5%) |
| *Ethnic identification by respondents* |  |  |
| Hispanic | 2/84 (2%) | 3/113 (3%) |
| Non-Hispanic | 74/84 (88%) | 100/113 (88%) |
| Declined | 8/84 (10%) | 10/113 (9%) |
| *Insurance Status reported by respondents* |  |  |
| Commercial dental insurance | 31/84 (37%) | 49/113 (43%) |
| Medicaid | 31/84 (37%) | 46/113 (41%) |
| Sliding Fee Discount (SFD) Program/FHC-M coverage | 10/84 (12%) | 13/113 (12%) |
| Self-Pay | 12/84 (14%) | 5/113 (4%) |
| *Transportation Status* |  |  |
| *Transportation to E-NTDC or last dental visit* |  |  |
| Own/family vehicle | 67/82 (82%) | 96/113 (85%) |
| Got a ride | 8/82 (10%) | 13/113 (11%) |
| Medical transport vehicle | 6/82 (7 %) | 3/113 (3%) |
| Bus | 1/82 (1%) | 1/113 (1%) |
| *Generally, I have transportation to dental appointments* |  |  |
| Yes | 72/81 (89%) | 102/112 (91%) |
| No | 5/81 (6%) | 4/112 (4%) |
| Sometimes | 4/81 (4%) | 6/112 (5%) |
| Knowledge-based Questions Responses | ***Cases*** | ***Controls*** |
| *Dentists’ guidance on tooth brushing frequency* |  |  |
| Once daily | 12/84 (14%) | 2/113 (2%) |
| Twice daily | 35/84 (42%) | 66/113 (58%) |
| After each meal | 36/84 (43%) | 41/113 (36%) |
| No recommendation or ‘Only when food gets stuck’ | 1/84 (1%) | 4/113 (3.5%) |
| *Dentists’ guidance on frequency of dental visits* | **Cases** | **Controls** |
| Once yearly | 26/82 (32%) | 28/112 (25%) |
| Twice yearly | 41/82 (50%) | 70/112 (62.5%) |
| More than twice yearly | 5/82 (6%) | 4/112 (3.5%) |
| As needed, when dental issues occur | 8/82 (10%) | 8/112 (7%) |
| There are no current guidelines | 2/82 (2%) | 3/112 (3%) |
| *Oral disease can worsen diabetes, heart or kidney disease* |  |  |
| True | 71/82(86.5%) | 101/112 (90%) |
| False | 3/82 (3.5%) | 0 |
| Don’t know | 8/82 (10%) | 11/112 (10%) |
| *Brushing teeth at least once daily is important* |  |  |
| True | 81/82 (99%) | 109/111 (98%) |
| False | 1/82 (1%) | 1/111 (1%) |
| Don’t know | 0 | 1/111 (1%) |
| *Regular dental visits can prevent pain; costly dental visits* |  |  |
| True | 81/82 (99%) | 107/111 (96%) |
| False | 0 | 2/111 (2%) |
| Don’t know | 1/82 (1%) | 2/111 (2%) |
| *Some oral infections can be life threatening* |  |  |
| True | 75/81 (93%) | 101/111 (91%) |
| False | 2/81 (2%) | 0 |
| Don’t know | 4/81 (5%) | 10/111 (9%) |
| *Dental visits are only needed if your mouth is unhealthy* |  |  |
| True | 6/81 (7.5%) | 4/108 (4%) |
| False | 73/81 (90%) | 104/108 (96%) |
| Don’t know | 2/81 (2.5%) | 0 |
| *Dental disease transfers across generations* |  |  |
| True | 14/79 (18%) | 14/111 (12.5%) |
| False | 40/79 (50.5%) | 73/111 (66%) |
| Don’t know | 25/79 (3.5%) | 24/111 (21.5%) |
| Patient-reported dental hygiene practices | **Cases** | **Controls** |
| *Frequency of tooth brushing* |  |  |
| Once daily | 30/79 (38%) | 41/111 (37%) |
| Twice daily | 34/79 (43%) | 59/111 (51%) |
| More frequently than twice daily | 8/79 (10%) | 10/111 (9%) |
| Not daily | 7/79 (9%) | 3/111 (3%) |
| *Frequency of dental checkups* |  |  |
| Once annually | 17/82 (21%) | 27/112 (24%) |
| Twice annually | 28/82 (34%) | 68/112 (61%) |
| More than twice a year | 5/82 (6%) | 14/112 (12%) |
| Not annually *or* only as needed | 30 (37%) | 3/112 (3%) |
| Family history of dental access | Cases | Controls |
| Attended dental visits only if dental issues arose | 21/82 (27%) | 36/112 (32%) |
| Did not like going to the dentist | 6/82 (7%) | 14/112 (12.5%) |
| Lost most of their teeth by age 50 years | 26/82 (32%) | 20/112 (18%) |
| Took me for dental visits at least once yearly | 35/82 (43%) | 65/112 (68%) |
| Encouraged me to regularly brush my teeth | 45/82 (55%) | 66/112 (59%) |
| Subject opinion regarding dental access | **Cases** | **Controls** |
| *Subject agreement with the following statements:* |  |  |
| Teeth are not worth saving | 1/83 (1%) | 0 |
| I would opt to have a painful tooth pulled rather than save it | 12/83 (14%) | 2/111 (2%) |
| Dental visits are only necessary for dental emergencies | 13/83 (16%) | 2/111 (2%) |
| I would schedule a dental visit only when pain relievers don’t work | 3/83 (4%) | 1/111 (1%) |
| I think seeing a dentist once a year is important | 69/83 (83%) | 107/111 (96%) |
| *How I would feel about attending a dental visit tomorrow:* |  |  |
| Anticipate that it will be fairly pleasant experience | 27/82 (33%) | 40/112 (36%) |
| My feelings would be neutral | 19/82 (23%) | 40/112 (36%) |
| I would feel slightly uneasy | 18/82 (22%) | 21/112 (19%) |
| I would be anxious about dental pain | 11/82 (13%) | 9/112 (8%) |
| I am anxious about having dental procedures | 1/82 (1%) | 0 |
| I would rather skip the appointment | 4/82 (4%) | 1/112 (1%) |
| *If I had dental pain, I would most likely:* |  |  |
| Take a pain medication and wait to see if it resolves | 18/82 (22%) | 15/110 (14%) |
| Take a pain medication and make an appointment | 34/82 (41%) | 60/110 (55%) |
| Make an emergency dental visit | 24/82 (29%) | 32/110 (29%) |
| Make an emergency medical visit | 3/82 (3%) | 0 |
| Other | 3/82 (3%) | 3/110 (3%) |

**Supporting Information 1**:

**Appendix C1:** The final survey tool distributed to ED-NTDC cases presenting at FHC-M dental centers during the study window from January 1, 2019, through May 19, 2019, is shown.

**Supporting Information 2:**

**Appendix C2:** The final survey tool distributed to age-range, gender-matched, and FHC-M dental center-matched controls attending a scheduled during the study’s temporal window from January 1, 2019, through May 19, 2019, is shown.

**Supporting Information 3:**

**Appendix C3:** The survey tool assessment questionnaire is shown. The questionnaire was completed by 10 anonymous volunteers during beta testing of the case/control surveys following a dental visit at an FHC-M dental center in Marshfield WI.

| **Appendix C3:**  **Summary of responses to survey tool assessment questionnaire following completion of the survey assessment tool by the anonymous respondent during initial piloting** | | |
| --- | --- | --- |
| **Did you find any of the questions to be:** | **yes** | **no** |
| **Too personal** | 1 | 9 |
| **Too sensitive** | 0 | 10 |
| **Too stressful** | 0 | 10 |
| **Were any questions confusing or difficult for you to understand?** | 0 | 10 |
| **If yes, please indicate question number(s)** | (none reported) | |

**Supporting information 3**

**Table D:** Observation of frequency of ED-NTDC visits across FHC-M dental centers during the observational window of the study. showed some seasonal variability when compared to the post-observational period through the remainder of 2019.

| Center | Jan | Feb | March | April | May | June | July | Aug | Sept | Oct | Nov | Dec |
| --- | --- | --- | --- | --- | --- | --- | --- | --- | --- | --- | --- | --- |
| Black River Falls | 78 | 80 | 85 | 71 | 75 | 101 | 145 | 105 | 127 | 143 | 102 | 138 |
| Chippewa | 414 | 337 | 255 | 315 | 254 | 355 | 410 | 432 | 270 | 410 | 368 | 323 |
| Lady Smith | 154 | 143 | 134 | 133 | 121 | 140 | 180 | 116 | 120 | 171 | 127 | 122 |
| Marshfield | 81 | 67 | 69 | 103 | 106 | 155 | 130 | 114 | 137 | 140 | 127 | 132 |
| Medford | 49 | 33 | 38 | 50 | 59 | 72 | 93 | 112 | 124 | 115 | 133 | 125 |
| Menomonie | 124 | 87 | 114 | 129 | 159 | 177 | 231 | 201 | 220 | 222 | 173 | 222 |
| Neillsville | 59 | 47 | 61 | 67 | 68 | 88 | 100 | 72 | 70 | 78 | 56 | 77 |
| Park Falls | 77 | 79 | 66 | 51 | 59 | 52 | 119 | 102 | 82 | 96 | 61 | 85 |
| Rhinelander | 176 | 103 | 128 | 173 | 175 | 189 | 199 | 194 | 229 | 234 | 168 | 201 |
| Rice Lake | 157 | 140 | 144 | 122 | 102 | 153 | 205 | 221 | 159 | 212 | 164 | 162 |
| Total | 1369 | 1116 | 1094 | 1214 | 1178 | 1482 | 1812 | 1669 | 1538 | 1821 | 1479 | 1587 |

**Supporting information 3**

**Table E:** Depicts the ratio of ED-NTDC cases seen at each FHC-M dental center during the ~5-month observational window of the study and the ~7 post-study months of 2019 juxtaposed to clinical practice days (CPD) logged monthly at each of the dental centers throughout the year. Ratios indicate a correlation between increased CPD and increased capacity to treat ED-NTDC cases in the post-study window. These data support high rates of ED-NTDC presenting to FHC-M service area and the high need for services to a population with high dental disparity access.

| **Table E: Ratio of #ED-NTDC cases/Clinical Practice Days (CPD) during and post study window, 2019** | | | | | | |
| --- | --- | --- | --- | --- | --- | --- |
| ***FHC-M Dental Center*** | **5 months**  **Jan-May**  **Case count** | **5 months**  **Jan-May Total CPD** | **5 months**  **Jan-May ratio of cases/CPD** | **7 months June-Dec**  **Case count** | **7 months**  **June-Dec**  **Total CPD** | **7 months**  **June-Dec. ratio of cases/CPD** |
| *Black River Falls* | 389 | 631 | 0.62 | 861 | 799 | 1.08 |
| *Chippewa Falls* | 1575 | 1161 | 1.36 | 2568 | 1640 | 1.57 |
| *Ladysmith* | 685 | 587 | 1.17 | 976 | 757 | 1.29 |
| *Marshfield* | 426 | 667 | 0.64 | 935 | 1052 | 0.89 |
| *Medford* | 229 | 891 | 0.26 | 774 | 1089 | 0.71 |
| *Menomonie* | 613 | 692 | 0.89 | 1446 | 936 | 1.54 |
| *Neillsville* | 302 | 442 | 0.68 | 541 | 485 | 1.12 |
| *Park Falls* | 332 | 432 | 0.77 | 597 | 465 | 1.28 |
| *Rhinelander* | 755 | 687 | 1.10 | 1414 | 907 | 1.56 |
| *Rice Lake* | 665 | 811 | 0.82 | 1275 | 993 | 1.28 |
| *All Centers* | 5971 | 7001 | 0.85 | 11387 | 9123 | 1.25 |
